# Total knee arthroplasty for knee osteoarthritis associated with abnormal patellar tendon deformity: a case report

**DOI:** 10.1093/jscr/rjae102

**Published:** 2024-03-06

**Authors:** Satoshi Miyamoto, Shin Sasaki, Hiroyuki Kojin, Ken Okazaki

**Affiliations:** Department of Orthopaedic Surgery, Kohsei Chuo General Hospital, 1-11-7 Mita Meguro-ku, Tokyo 153-8581, Japan; Department of Orthopaedic Surgery, Tokyo Women’s Medical University Yachiyo Medical Center, 477-96 Owada-Shinden, Yachiyo-shi, Chiba 276-8524, Japan; Department of Orthopaedic Surgery, Kohsei Chuo General Hospital, 1-11-7 Mita Meguro-ku, Tokyo 153-8581, Japan; Department of Clinical Quality and Medical Safety Management, University of Yamanashi Hospital, 1110 Shimokato, Chuoshi, Yamanashi 409-3898, Japan; Department of Orthopaedic Surgery, Tokyo Women’s Medical University, 8-1 Kawadacho, Shinjuku-ku, Tokyo 162-8666, Japan

**Keywords:** patellar tendon deformity, valgus knee deformity, total knee arthroplasty

## Abstract

There have been no earlier reports of knee osteoarthritis with valgus knee deformity in which the patellar tendon infiltrates the tibial bone marrow instead of attaching to the tibial tubercle. This case report describes a total knee arthroplasty (TKA) performed for the treatment of a primary knee osteoarthritis resulting from a valgus knee joint position attributed to an abnormality of the patellar ligament attachment. During a TKA, the tendon tissue in the tibial medullary canal interfered with the reamer used to prepare for the stem extensions needed to improve the fixation of the component on the tibia, which had a cortical defect. The arthroplasty succeeded, and good clinical results have been maintained over the 3 years since the surgery. Surgeons should consider careful preoperative examinations by magnetic resonance imaging or CT when an abnormal bone defect is observed at the tibial tubercle on plain X-ray images.

## Introduction

The patellar tendon attachment extends to the nonarticular part of the patella surface, up to the distal edge of the patellar articular surface, and to the distal attachment to the tibial tubercle [1]. Patellar tendonitis is caused by the dissection of the attachment located between the patella and patellar tendon [2]. To the best of our knowledge, there have been no earlier reports of knee osteoarthritis with valgus knee deformity in which the patellar tendon infiltrates the tibial bone marrow instead of attaching to the tibial tubercle.

This case report describes a total knee arthroplasty (TKA) performed for the treatment of a primary knee osteoarthritis resulting from a valgus knee joint position attributed to an abnormality of the patellar ligament attachment.

## Case report

A 73-year-old Asian woman had been undergoing conservative treatment, but the pain gradually worsened, and she was referred to our hospital for TKA.

The patient had no specific history of trauma to the knee but had undergone a surgery for gastric cancer 3 years before.

**Figure 1:**
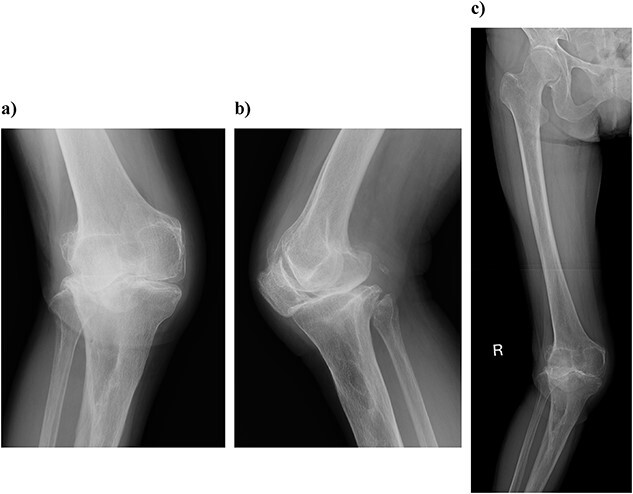
Plain radiography at the patient’s first visit: (a) standing front, (b) lateral side and (c) thigh, standing, front, full length. Valgus knee osteoarthritis and well-defined osteoporosis near the central part of the proximal tibia can be seen, and the rough surface of the tibia has been worn away.

**Figure 2:**
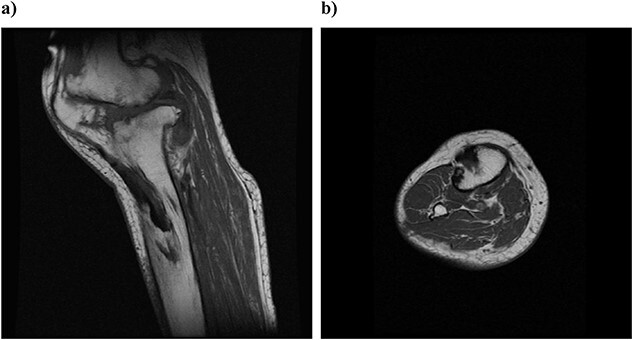
Preoperative simple magnetic resonance imaging: (a) lateral side and (b) axial morphism. T1- and T2-weighted images continuous from the patellar tendon attached to the patella both show continuity from the low signal area to the inside of the tibial bone marrow.

On physical examination, the patient extension and flexion were − 20° and 130°. A defect of the tibial tubercle was observed. The Knee Society (KS) score was 44 points, and the Knee Society function (KS-F) score was 70 points. A plain radiography showed valgus knee osteoarthritis assessed as Kellgren–Lawrence grade 4. In addition, a well-defined translucent image of bone appeared near the central part of the proximal tibia and no tibial tubercle could be observed (Fig. 1). Magnetic resonance imaging (MRI) of the right knee joint showed the patellar tendon appeared from the attachment on the patella with continuity to the tibial bone marrow in T1- and T2-weighted imaging (Fig. 2).

TKA was selected as the treatment to improve her symptoms.

Surgery was performed under general anesthesia, the surgical limb position was supine, and no tourniquet was used. A midline incision of about 20 cm was made, and the joint was reached by the parapatellar approach.

**Figure 3:**
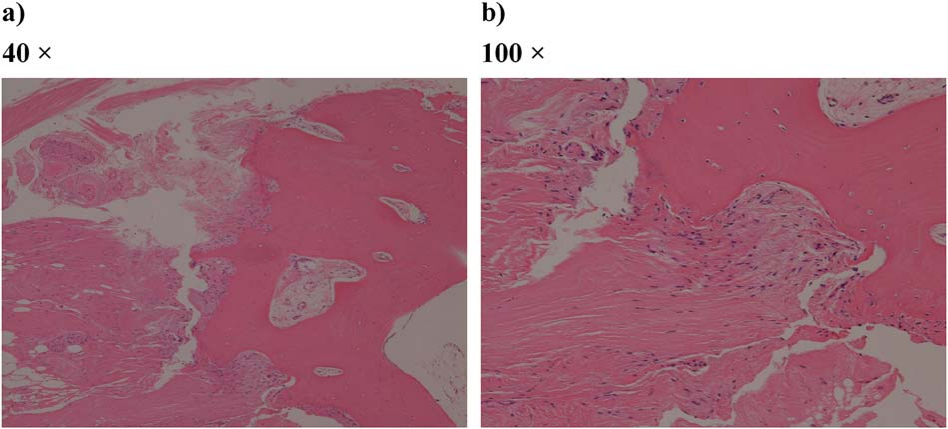
Pathologic specimen from surgery (hematoxylin–eosin staining): (a) 40 times and (b) 100 times. Bone tissue and fibrous tissue are both conspicuous, and ligament-like tissue is partially observed.

**Figure 4:**
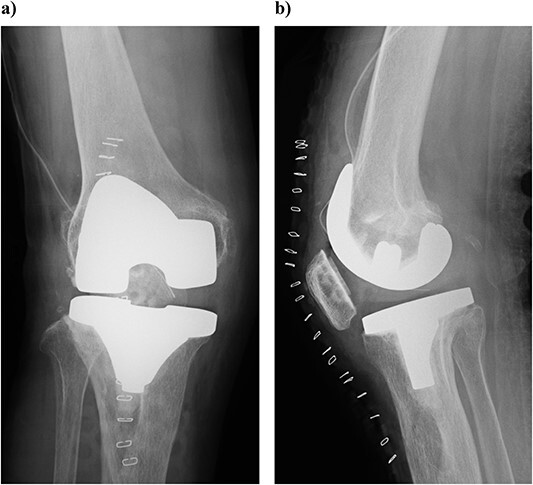
Plain radiography immediately after the surgery: (a) front and (b) lateral side. A slight dilation of the medial joint space is observed.

The knee was replaced with a Scorpio NRG PS type system (Stryker Orthopedics, Mahwah, New Jersey). The distal femur was cut with an intramedullary rod guided at 6° valgus. The tibia was cut with an extra medullary rod 10 mm from the medial joint surface, and a cutting guide was installed at a posterior inclination of 0°. The tibia was reamed, once a depth of about 30 mm was reached, the reamer could not be easily advanced. Once the tip of the reamer was confirmed, the tendon fiber was wrapped around the remar (Fig. 3). When palpated from the anterior lateral side of the tibia, the invasion of the patellar tendon into the bone marrow was confirmed. The patella was cut 8 mm from the articular surface. In addition, poor tracking of the patella caused lateral release. All of the prostheses were fixed using bone cement. Slight dilation of the medial joint space was observed at the time of closure, and the fascial was sutured with an overlapping stitch (Fig. 4). The operation time was 190 minutes and the amount of intraoperative bleeding was 238 ml.

On postoperative Day 1, the knee joint was fixed with a hinged knee brace and rehabilitation was started. The continuous passive motion (CPM) was started twice daily. The hinged knee brace was removed on Day 33 and the practice of climbing stairs was started on Day 36.

**Figure 5:**
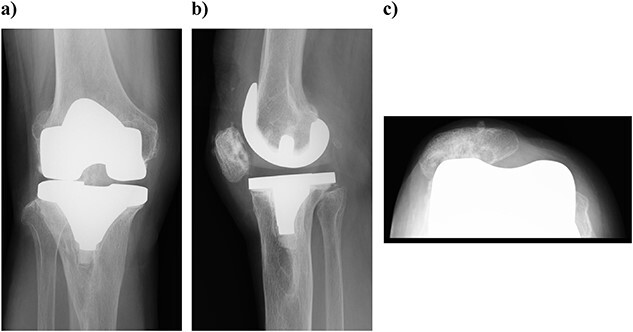
Outcome at 3 years after surgery on plain radiography: (a) standing front and (b) side. The patella is laterally subluxated in a slight valgus position.

At 3 years postoperatively, the patient could walk independently. Her extension and flexion were − 5° and 120°, respectively, her KS score was 83 points, and her KS-F score was 90 points. Radiography showed a slight valgus position, with some lateral subluxation of the patella, but the patient reported no problems with her activities of daily living (Fig. 5).

## Discussion

No reports in the literature before now have described invasions of the patellar tendon into the tibial bone marrow.

In this TKA of the valgus knee, we planned to lengthen the stem of the tibia because the balance between the medial and lateral sides could not be achieved, and because the patellar tendon entered the tibial bone marrow. As the patellar tendon was found to be involved at that depth, the extension of the stem was abandoned. If we had taken a CT to predict the occupation rate of the patellar tendon in the medullary cavity, we might have had the option of changing the direction of the reaming or taking some other countermeasure.

Many reports have described the length of the patellar tendon. Gloria *et al*. [3] reported that the patellar tendon was clearly shorter on the left in many individuals, and that no sex differences were evident.

Guijin *et al*. [4] reported that the lateral approach was useful for valgus knee. We believe that the lateral approach should be considered when the medial approach does not provide a sufficient field of view.

TKA of the valgus knee is considered to be prone to patellar subluxation. Kawaguchi *et al*. [5] suggested that the condition be prevented by considering differences in postoperative rotational kinematics when selecting the implant and planning the surgical technique. Detailed preoperative planning, careful selection of the mechanical implants and the engagement of an experienced surgeon may be useful in preventing postoperative patellar subluxation.

Cheng *et al*. [6] reported that primary repair of an MCL injury during TKA could provide an excellent clinical outcome with good stability. By sufficiently evaluating the MCL during the operation we report here, we expected the medial laxity observed intraoperatively would improve.

If a defect is found at the tibial tubercle on X-ray examination, the surgeon should consider evaluating the soft tissue on MRI when planning the surgery. If anatomical abnormalities in bone morphology are found, the shape of the bone must also be evaluated on CT. Based on the image evaluations, it was necessary to plan for options, in preparation for the TKA.

In conclusion, we performed a TKA for a case valgus knee osteoarthritis thought to have been caused by an abnormality in the attachment of the patellar ligament. Surgeons should consider careful preoperative examinations.

## Data Availability

The data presented in this study are available from the corresponding author, when requested for reasonable purposes.

## References

[1] Soames RW . The knee joint. In: Williams PL (ed). Grays Anatomy, 38th edn. 1995, 697–709.

[2] Basso O , Johnson DP, Amis AA. The anatomy of the patellar tendon. Knee Surg Sports Traumatol Arthrosc 2001;9:2–5. 11269580 10.1007/s001670000133

[3] Gloria M , Hohenberger GM, Dreu M, et al. Patellar tendon length is associated with lower extremity length but not gender. Indian J Orthop 2020;54:352–7. 32399156 10.1007/s43465-020-00046-1 PMC7205968

[4] Xu G , Fu X, Tian P, et al. The lateral and medial approach in total arthroplasty for valgus knee: a meta-analysis of current literature. J Comp Eff Res 2020;9:35–44. 10.2217/cer-2019-0111. 31777265

[5] Kawaguchi K , Inui H, Taketomi S, et al. Rotational kinematics differ between mild and severe valgus knees in total knee arthroplasty. Knee 2021;28:81–8. 33310669 10.1016/j.knee.2020.10.010

[6] Jin C , Zhao JY, Santoso A, et al. Primary repair for injury of medial collateral ligament during total-knee arthroplasty. Medicine 2019;98:e17134. 31574814 10.1097/MD.0000000000017134 PMC6775350

